# Correlation between intracranial pressure monitoring for severe traumatic brain injury with hospital length of stay and discharge disposition: a retrospective observational cohort study

**DOI:** 10.1186/s13037-022-00350-9

**Published:** 2022-12-29

**Authors:** Christopher W. Foote, Stephanie Jarvis, Xuan-Lan Doan, Jordan Guice, Bianca Cruz, Cheryl Vanier, Alejandro Betancourt, David Bar-Or, Carlos H. Palacio

**Affiliations:** 1 McAllen Medical Center, South Texas Health System, 301 W Expy 83, McAllen, TX 78503 USA; 2 Valley Health Systems, Graduate Medical Education, 2075 East Flamingo Rd, Las Vegas, NV 89119 USA; 3 Injury Outcomes Network (ION) Research, 501 East Hampden Ave, Englewood, CO 80113 USA; 4 Brooke Army Medical Center, 8551 Roger Brooke Dr, San Antonio, TX 78219 USA; 5 Touro University, School of Medicine, 874 American Pacific Dr, Henderson, NV 89014 USA

**Keywords:** Traumatic brain injury, Intracranial hypertension, Intracranial pressure monitor, Guideline

## Abstract

**Objectives:**

Intracranial pressure (ICP) monitoring is recommended for severe traumatic brain injuries (TBI) but some data suggests it may not improve outcomes. The objective was to investigate the effect of ICP monitoring among TBI.

**Methods:**

This retrospective observational cohort study (1/1/2015–6/1/2020) included severe TBI patients. Outcomes [discharge destination, length of stay (LOS)] were compared by ICP monitoring and were stratified by GCS (3 vs. 4–8), α < 0.05.

**Results:**

Of the123 patients who met inclusion criteria, 47% received ICP monitoring. There were baseline differences in the two groups characteristics, ICP monitored patients were younger (*p* = 0.02), had a subarachnoid hemorrhage less often (*p* = 0.04), and a subdural hematoma more often (*p* = 0.04) than those without ICP monitors. ICP monitored patients had a significantly longer median LOS (12 vs. 3, *p* < 0.01) than patients without monitoring. There was a trend towards more ICP monitored patients discharged home (40% vs. 23%, *p* = 0.06). Among patients with GCS = 3, ICP monitored patients had a longer LOS (*p* < 0.01) with no significant differences in discharge destinations. For those with a GCS of 4–8, ICP monitoring was associated with a longer LOS (*p* = 0.01), but fewer were discharged to a skilled nursing facility or long-term care (*p* = 0.01).

**Conclusions:**

For TBI patients, ICP monitoring was associated with an increased LOS, with no significant differences in discharge destinations when compared to those without ICP monitoring. However, among only those with a GCS of 4–8, ICP monitoring was associated with a decreased proportion of patients discharged to a skilled nursing facility or long-term acute care .

## Introduction

Severe traumatic brain injury (TBI) management focuses on preventing secondary insults [1]. The Brain Trauma Foundation severe TBI guideline recommends treatment guided by monitoring modalities including intracranial pressure (ICP) monitors [2]. They state that Level 2B evidence shows ICP monitoring may reduce mortality rates [2]. The Brian Injury Guideline, recommends that severe TBIs, categorized as Brain Injury Guideline 3, receive repeat imaging, a neurosurgeon consultation, and are admitted to the hospital, but have no ICP monitoring recommendations [3].

There is conflicting evidence on ICP monitoring efficacy for improving morbidity and mortality [4–11]. Differing results may be attributable to subsequent treatments used due to ICP monitoring results [2]. ICP monitoring is recommended for patients with a Glasgow Coma Scale (GCS) ≤ 8 and an abnormal head computed tomography (CT) [2]. It is possible that patients with GCS = 3 may be skewing the mortality rate of ICP monitoring; one study of ICP monitored patients with GCS = 3 reported a mortality rate of 49%, another of ICP monitored patients with GCS 3–8 reported a mortality rate of 24% [9, 12]. Although many studies examined the effect of ICP placement, there is little evidence on the role of GCS. The study purpose was to 1) compare the effect of ICP monitoring among TBI patients on outcomes providing additional evidence on its efficacy, and 2) to further stratify results by GCS. The hypothesis was that there would be no differences in outcomes by ICP monitoring overall, nor for those with a GCS = 3, but ICP placement would improve outcomes for those with a GCS of 4–8.

## Methods

This was a retrospective observational cohort study comparing severe TBI patients who received ICP monitoring to those who did not at a Level II trauma center. Severe TBI was defined as having an admission GCS ≤ 8 and Brain Injury Guidelines 3 classification per imaging characteristics [subdural hematoma (SDH) > 8 mm, epidural hematoma (EDH) > 8 mm, intra-parenchymal hematoma (IPH) > 8 mm and multiple locations, scattered subarachnoid hemorrhage (SAH), and intraventricular hemorrhage (IVH)] [3]. Included patients were enrolled consecutively over a 5.5-year period starting on 01/01/2015 and ending on 06/01/2020. Additional selection criteria were as follows. Blunt and penetrating injuries were included, burn patients were excluded. Patients could have suffered an isolated TBI or poly-trauma. There were no exclusions applied based on age. The Western Investigational Review Board approved of this study with a waiver of consent. Data were collected from the trauma registry and electronic medical records. ICP monitors were placed at the trauma surgeon’s discretion.

The primary outcome was discharge destination, which was categorized as: in-hospital mortality, home, rehabilitation, or skilled nursing facility/long-term acute care. Secondary outcomes included hospital length of stay (LOS), intensive care unit length of stay (intensive care unit LOS), total ventilator days, and pneumonia. Outcomes were collected from the trauma registry. Fisher’s exact test and Chi-squared test were used to compare categorical or dichotomous data, which were summarized as proportion (count). Kruskal Wallis or Student’s t-tests were used to compare continuous variables based on the distribution of the data. Continuous variables were summarized as median with interquartile range, when non-parametric, and means with standard deviation, when parametric. In a post-hoc analysis of these outcomes, the average power was 85%. Because of the effect of GCS on mortality in previous studies, a stratified analysis was conducted based on the GCS (3 vs. 4–8). A significance level of α < 0.05 and Statistical Analysis System v9.4 (Cary, North Carolina) were used for all analyses.

## Results

A total of 123 patients met the selection criteria. Of those 47% received ICP monitoring and 53% did not. Overall, the median age was 43 and 34% were female, Table 1. Regarding the patient’s demographics, those with ICP monitoring were significantly younger (*p* = 0.02) than those without ICP monitoring. Other demographics were similar between groups; there were no differences in gender (*p* = 0.96), race (*p* = 0.20), or ethnicity (*p* > 0.99). The severity of patients’ injuries measured through the GCS (*p* = 0.48) and Injury Severity Scale (ISS) (*p* = 0.91) were also comparable by ICP monitoring.

**Table 1:** Demographics and Baseline Characteristics

	No ICP Monitor ***n*** = 65	ICP Monitor ***n*** = 58	***p***	Total ***n*** = 123
Age, Median (IQR)	54.0 (29.0, 71.0)	34.0 (22.0, 65.0)	**0.02**	42.5 (25.0, 69.0)
Female, % (n)	33.8% (22)	32.7% (19)	0.96	34.2% (42)
Race, % (n)				
White	89.5% (17)	100% (23)	0.20	95.2% (40)
Other	10.5% (2)	0% (0)		4.8% (2)
Hispanic or Latino, % (n)	88.9% (16)	86.4% (19)	> 0.99	87.5% (35)
GCS, Median (IQR)	3.0 (3.0, 6.0)	3.0 (3.0, 6.0)	0.48	3.0 (3.0, 6.0)
3	60.0% (36)	51.7% (30)	0.85	55.9% (66)
4	8.3% (5)	8.6% (5)		11.0% (13)
5	3.3% (2)	5.2% (3)		4.2% (5)
6	8.3% (5)	13.8% (8)		11.0% (13)
7	8.3% (5)	12.1% (7)		10.2% (12)
8	11.7% (7)	8.6% (5)		10.2% (12)
SBP, Mean (SD)	145.9 (43.8)	142.3 (32.5)	0.62	144.1 (38.4)
ISS, Median (IQR)	25.0 (17.0, 28.0)	25.0 (17.0, 27.0)	0.91	25.0 (17.0, 27.0)

Bold numbers indicate statistical significance at *p* < 0.05

*ICP* Intracranial pressure, *GCS* Glasgow coma scale, *SBP* systolic blood pressure, *ISS* Injury Severity Score, *IQR* interquartile range, *SD* standard deviation

There were observed differences in the hemorrhage type by ICP monitoring. There was a lower proportion of ICP monitored patients with a SAH (50% vs 66%, *p* = 0.04), and a higher proportion with a SDH (74% vs. 54%, *p* = 0.04), when compared to those without ICP monitoring, Table 2. Other types of bleeds were similar. There was no difference in the proportion of skull fractures (*p* = 0.14). There was a significantly higher proportion of ICP monitored patients who had a craniotomy (48% vs. 12%, *p* < 0.0001), craniectomy (38%, vs 3%, *p* < 0.0001), tracheostomy (40% vs. 15%, *p* = 0.004), or gastrointestinal tube placed (40% vs. 17%, *p* = 0.01) when compared to those without ICP monitoring. Of the ICP monitored patients, 56% needed an external ventricular drain, 22% needed a bolt, and 5% needed a different type of drain.

**Table 2:** Injury Characteristics and Procedures

	No ICP Monitor ***n*** = 65	ICP Monitor ***n*** = 58	***P***	Total ***n*** = 123
ICH Type, % (n)				
SAH	66.2% (43)	50.0% (29)	**0.04**	58.5% (72)
SDH	53.8% (35)	74.1% (43)	**0.04**	63.4% (78)
EDH	13.8% (9)	5.2% (3)	0.13	9.8% (12)
IPH	24.6% (16)	31.0% (18)	0.50	27.6% (34)
IVH	9.2% (6)	10.3% (6)	0.88	9.8% (12)
Extra-axial	6.2% (4)	8.6% (5)	0.74	7.3% (9)
Skull Fracture, % (n)	52.3% (34)	67.2% (39)	0.14	59.5% (73)
In-hospital Procedures, % (n)				
Craniotomy	12.3% (8)	48.3% (28)	**< 0.0001**	29.3% (36)
Craniectomy	3.1% (2)	37.9% (22)	**< 0.0001**	19.5% (24)
Reimplant Skull	0% (0)	1.7% (1)	0.48	0.8% (1)
Tracheostomy	15.4% (10)	39.7% (23)	**0.004**	26.8% (33)
G-tube	16.9% (11)	39.7% (23)	**0.01**	27.6% (34)
IVC Filter	4.6% (3)	5.2% (3)	> 0.99	4.9% (6)
ICP Monitor Characteristics, % (n)				
EVD	N/A	55.9% (33)	N/A	26.8% (33)
Bolt	N/A	22.2% (13)	N/A	10.6% (13)
Drain	N/A	5.1% (3)	N/A	2.4% (3)

Bold numbers indicate statistical significance at *p* < 0.05

*ICP* Intracranial pressure, *ICH* intracranial hemorrhage, *SAH* subarachnoid hemorrhage, *SDH* subdural hemorrhage, *EDH* epidural hemorrhage, *IPH* intraparenchymal hemorrhage, *IVH* intraventricular hemorrhage, *IVC* inferior vena cava, *CT* computed tomography, *EVD* external ventricular drain, *N/A* not applicable

There was a trend towards having a significantly higher proportion of ICP monitored patients discharged home (40% vs 23%, *p* = 0.06) when compared to patients without ICP monitoring, Table 3. Other discharge destinations, including mortality, were comparable. A higher proportion of ICP monitored patients had pneumonia (28% vs. 3%, *p* = 0.0002) than patients without ICP monitoring. There were no deaths among patients with pneumonia, in fact patients with pneumonia who had ICP monitoring were more often discharged home than those without ICP monitoring (26% vs. 9%, *p* = 0.01). ICP monitored patients had a significantly longer intensive care unit LOS (11 vs 3, *p* = 0.0001), total ventilator days (6 vs 2, *p* < 0.0001), and LOS (12 vs 3, *p* < 0.0001) than patients without ICP monitoring.

**Table 3:** Complications & Outcomes

	No ICP Monitor ***n*** = 65	ICP Monitor ***n*** = 58	***p***	Total ***n*** = 123
Complications, % (n)				
Pressure Ulcer	4.7% (3)	3.4% (2)	> 0.99	4.0% (5)
Pneumonia	3.1% (2)	27.6% (16)	**0.0002**	14.6% (18)
ICP Complication	N/A	6.8% (4)	N/A	N/A
Intensive Care Unit LOS, Median (IQR)	3 (1, 9)	11 (3, 19)	**0.0001**	5 (2, 14)
Ventilator Days, Median (IQR)	2 (1, 3)	6 (3, 16)	**< 0.0001**	3 (1, 9)
LOS, Median (IQR)	3 (2, 11)	12 (5, 25)	**< 0.0001**	6 (2, 20)
Discharge Destination, % (n)				
In-hospital Mortality	43.1% (28)	34.5% (20)	0.26	39.0% (48)
Home	23.1% (15)	39.7% (23)	0.06	30.9% (38)
Skilled Nursing Facility / Long-Term Acute Care	26.2% (17)	17.2% (10)	0.20	22.0% (27)
Rehabilitation	6.2% (4)	10.3% (6)	0.52	8.1% (10)

ICP complications included: kinking, malfunctioning, or dislodging. Bold numbers indicate statistical significance at *p* < 0.05

*ICP* intracranial pressure, *ICULOS* intensive care unit length of stay, *LOS* hospital length of stay, *IQR* interquartile range, *N/A* not applicable

In a stratified analysis of only patients with a GCS = 3, ICP monitoring was still associated with a significantly higher rate of pneumonia (*p* = 0.01), a significantly longer intensive care unit LOS (*p* = 0.002), ventilator days (*p* = 0.0002), and LOS (*p* = 0.001) when compared to patients without ICP monitoring, Table 4. Among patients with a GCS of 4–8, ICP monitoring was still associated with a significantly higher rate of pneumonia (*p* = 0.03), and longer LOS (*p* = 0.01); but intensive care unit LOS (*p* = 0.08) and ventilator days (*p* = 0.07) were only trending towards being significantly longer when compared to those without ICP monitoring. There was a significantly lower proportion of patients with a GCS of 4–8 and ICP monitoring who were discharged to a skilled nursing facility or long-term acute care when compared to those without ICP monitoring (*p* = 0.01) and there was also a trend towards a higher proportion of ICP monitored patients being discharged home (*p* = 0.09) than patients without ICP monitoring.

**Table 4:** Outcomes Stratified by GCS

	GCS = 3			GCS 4–8		
	No ICP Monitoring ***n*** = 36	ICP Monitoring ***n*** = 30	***p***	No ICP Monitoring ***n*** = 24	ICP Monitoring ***n*** = 28	***p***
Complications, % (n)						
Pressure Ulcer	2.8% (1)	3.3% (1)	> 0.99	8.3% (2)	3.6% (1)	0.58
Pneumonia	2.8% (1)	26.7% (8)	**0.01**	4.1% (1)	28.6% (8)	**0.03**
ICP Complication	N/A	6.7% (2)	N/A	N/A	7.1% (2)	N/A
Intensive Care Unit LOS, Median (IQR)	2 (1, 5)	8 (3, 16)	**0.002**	5 (1, 16)	12 (4, 21)	0.08
Ventilator Days, Median (IQR)	2 (1, 3)	6 (3, 14)	**0.0002**	2 (1, 6)	6 (3, 17)	0.07
LOS, Median (IQR)	3 (2, 6)	10 (4, 21)	**0.001**	6 (2, 18)	16 (7, 27)	**0.01**
Discharge Destination, % (n)						
Death	63.9% (23)	43.3% (13)	0.09	16.7% (4)	21.4% (6)	0.74
Home	13.9% (5)	23.3% (7)	0.32	33.3% (8)	57.1% (16)	0.09
Skilled Nursing Facility or Long-Term Acute care	19.4% (7)	23.3% (7)	0.70	41.7% (10)	10.7% (3)	**0.01**
Rehabilitation	2.8% (1)	10.0% (3)	0.32	8.3% (2)	10.7% (3)	> 0.99

ICP complications included: kinking, malfunctioning, or dislodging. Bold numbers indicate statistical significance at *p* < 0.05

*GCS* Glasgow Coma Scale, *ICP* intracranial, *ICULOS* intensive care unit, *LOS* hospital length of stay, *IQR* interquartile range, *N/A* not applicable

## Discussion

Based on this observational study, TBI patients with ICP monitoring may have an extended intensive care unit LOS, LOS, ventilator days and a higher rate of pneumonia. Discharge destinations were generally improved with ICP monitoring but this association was dependent on GCS. For patients with a GCS of 4–8 there was a significantly lower proportion of ICP monitored patients discharged to a skilled nursing facility or long-term acute care and a trend towards a higher proportion of ICP monitored patients discharged home when compared to those without ICP monitoring. Whereas for those with a GCS = 3, there were no significant associations with the discharge destination.

Like this study, previous studies found ICP monitoring did not impact mortality [10, 11, 13]. Others observed lower mortality rates with ICP monitoring [5, 6]. Some studies have found a higher mortality with ICP monitoring [8, 9, 14]. Lane et al. found that after controlling for ISS and mechanism, ICP monitoring was associated with improved survival [4]. In this study there were no differences in the initial ISS, GCS, or mortality.

In contrast to this study, Tang et al. found a significantly lower proportion of ICP monitored patients discharged home [9]. While there was an association between patients with a GCS of 4–8 and improved discharge destinations with ICP monitoring, there were no differences observed among patients with GCS = 3. Lane et al. reported that the head abbreviated injury scale score was significantly associated with in-hospital mortality, where patients with the worst scores were more likely to die in-hospital [4]. In a study of pediatric patients who had an ICP placed, GCS = 3 also significantly increased in-hospital mortality [15].

ICP monitoring was associated with a significantly longer LOS, intensive care unit LOS, and ventilator days in this study, which could be due to the Brain Trauma Foundation recommendations on further treatment based on ICP results [2]. Additional studies have observed longer stays and ventilator days with ICP monitoring [5, 7, 8, 11, 14]. While Haddad et al. saw no difference in LOS [13]. In this study, patients with GCS = 3 experienced significantly longer intensive care unit LOS, ventilator days, and LOS with ICP monitoring; whereas for those with a GCS of 4–8 there was no significant difference in the intensive care unit LOS or ventilator days, but a significantly longer LOS with ICP monitoring.

Two other studies also observed that patients with an ICP monitor were more likely to have pneumonia [11, 13]. One possible explanation for this could be the increased ventilator days, or increased use of invasive treatments among ICP monitored patients. Among the patients with pneumonia, there were no deaths.

There were notable differences between the groups. ICP monitored patients were younger, which has been previously reported [5, 8, 11, 14]. Like other studies, the majority of ICP monitored patients had neurosurgical interventions, likely due to recommendations that ICP monitoring be used to guide treatment [2, 5, 8, 14]. Other studies reported no difference in the hemorrhage type by ICP monitoring but in this study ICP monitored patients had significantly more SDHs and less SAHs [6, 9, 16].

## Limitations

This was a single-center retrospective study with a small sample size that limited the ability to conduct propensity matching or adjusted modeling and may not be generalizable. Factors related to the ICP monitor (indication, drainage volumes, etc.) were not collected. The procedure date/times were not collected so we could not determine if procedures occurred after ICP placement. There was no long-term follow-up. There was not a protocol followed by all treating physicians guiding ICP monitoring, therefore these results may not be generalizable to centers utilizing an existing protocol. Implementation of a protocol guiding ICP monitoring may result in different outcomes than seen in this study.

## Conclusions

This study observed that for TBI patients ICP monitoring may be associated with a higher rate of pneumonia, an increased intensive care unit LOS, ventilator days, and LOS, with no difference in the discharge destinations, including the mortality rate, when compared to those without ICP monitoring at a Level II trauma center without a protocol guiding ICP monitoring. While ICP monitoring may not improve outcomes across all patients, select patients may have improved discharge destinations, despite longer LOS. Among those with a GCS of 4–8, ICP monitoring was associated with a significant decrease in the proportion of patients discharged to a skilled nursing facility or long-term acute care when compared to those without an ICP monitor.

## Data Availability

The datasets generated and/or analyzed during the current study are not publicly available, as they were compiled from our own patient records, but data and coding methodology are available from the corresponding author on reasonable request.

## Abbreviations

TBI: Severe traumatic brain injury
ICP: Intracranial pressure
GCS: Glasgow Coma Scale
CT: Computed tomography
SDH: Subdural hematoma
EDH: Epidural hematoma
IPH: Intra-parenchymal hematoma
SAH: Subarachnoid hemorrhage
IVH: Intraventricular hemorrhage
LOS: Hospital length of stay, intensive care unit
ISS: Injury Severity Scale
